# Characterization of Chaotic Electroconvection near Flat Inert Electrodes under Oscillatory Voltages

**DOI:** 10.3390/mi10030161

**Published:** 2019-02-26

**Authors:** Jeonglae Kim, Scott Davidson, Ali Mani

**Affiliations:** 1 School for Engineering of Matter, Transport and Energy, Arizona State University, Tempe, AZ 85287, USA; jeokim@asu.edu; 2 Center for Turbulence Research, Stanford University, Stanford, CA 94305, USA; davscott@alumni.stanford.edu

**Keywords:** Electroconvection, AC electrokinetics, inert electrodes

## Abstract

The onset of electroconvective instability in an aqueous binary electrolyte under external oscillatory electric fields at a single constant frequency is investigated in a 2D parallel flat electrode setup. Direct numerical simulations (DNS) of the Poisson–Nernst–Planck equations coupled with the Navier–Stokes equations at a low Reynolds number are carried out. Previous studies show that direct current (DC) electric field can create electroconvection near ion-selecting membranes in microfluidic devices. In this study, we show that electroconvection can be generated near flat inert electrodes when the applied electric field is oscillatory in time. A range of applied voltage, the oscillation frequency and the ratio of ionic diffusivities is examined to characterize the regime in which electroconvection takes place. Similar to electroconvection under DC voltages, AC electroconvection occurs at sufficiently high applied voltages in units of thermal volts and is characterized by transverse instabilities, physically manifested by an array of counter-rotating vortices near the electrode surfaces. The oscillating external electric field periodically generate and destroy such unsteady vortical structures. As the oscillation frequency is reduced to $O(10^{-1})$ of the intrinsic resistor–capacitor (RC) frequency of electrolyte, electroconvective instability is considerably amplified. This is accompanied by severe depletion of ionic species outside the thin electric double layer and by vigorous convective transport involving a wide range of scales including those comparable to the distance *L* between the parallel electrodes. The underlying mechanisms are distinctly nonlinear and multi-dimensional. However, at higher frequencies of order of the RC frequency, the electrolyte response becomes linear, and the present DNS prediction closely resembles those explained by 1D asymptotic studies. Electroconvective instability supports increased electric current across the system. Increasing anion diffusivity results in stronger amplification of electroconvection over all oscillation frequencies examined in this study. Such asymmetry in ionic diffusivity, however, does not yield consistent changes in statistics and energy spectrum at all wall-normal locations and frequencies, implying more complex dynamics and different scaling for electrolytes with unequal diffusivities. Electric current is substantially amplified beyond the ohmic current at high oscillation frequencies. Also, it is found that anion diffusivity higher than cation has stronger impact on smaller-scale motions (≲0.1L).

## 1. Introduction

Aqueous electrolytes in contact with charge-selective surfaces are found in various biological systems and electrochemical applications. Such surfaces include ion-selective membrane, electrode and nanochannel, which have a net surface charge density. As a result, counterions (ions having charges opposite to the net surface polarity) are attracted toward the surface, while coions are repelled. Such redistribution of ions leads to the formation of electric double layers (EDL), which involve a charged fluid region near the interface screening the solid phase.

In most of these applications, externally applied electric fields are used to drive ions in electrolytes to achieve objectives such as chemical separation, purification or reaction. When an electric field drives counterions towards a charge-selective surface, the interaction of the electric field with adjacent charged fluid generates body forces that can result in a hydrodynamic instability called electroconvection [1,2]. One characteristic feature of electroconvective instability is increased mixing and scalar transport, facilitated by the arrays of large-scale counter-rotating vortex pairs. Recent studies have provided important evidence that enhanced hydrodynamic mixing is well correlated with overlimiting current measured at high applied voltages [3,4,5].

Prevalent to the onset of electroconvection are two other physical phenomena: concentration polarization and formation of an extended space charge region. The former is observed first, and is a consequence of two constraints. One constraint is blockage of coion flux to or from the interface due to selectivity of the solid boundary. The other constraint is system’s tendency towards bulk electroneutrality, thus maintaining equal local concentration of coions and counterions. These two constraints can be satisfied simultaneously only in the presence of concentration gradients such that diffusion fluxes can be comparable to electromigration fluxes [6]. This phenomenon, called concentration polarization, results in reduced concentration of both co- and counterions near the selective surface causing a transport-limited current, which cannot be exceeded with increased voltage, as long as bulk electroneutrality holds. When the applied voltage is further increased, bulk electroneutrality breaks down and a region dominated by counterions, called the extended space charge (ESC) region, is formed in the bulk adjacent to the EDL [6]. Unlike the diffuse charge region of the EDL, ions in the ESC are not Boltzmann distributed. Dynamics of ESC plays a significant role in electroconvection [1,2,7]

Following the theoretical work of Zaltzman and Rubinstein [2], electroconvective instability has been confirmed experimentally in settings involving charge-selective membranes [4,5]. In similar systems, the full nonlinear response has been theoretically investigated via direct numerical simulations (DNS) [8,9,10,11]. Typically, arrays of coherent vortical structures are observed to emerge near charge-selective surfaces. Beyond a threshold voltage, electroconvection is shown to lead to chaotic flows with structures involving a range of scales with broadbanded spectra [8,12], and ultimately with self-similar structures analogous to turbulent flows (despite their zero Reynolds number) [13]. Investigation of such regimes requires DNS of the fully-coupled Poisson–Nernst–Planck and Stokes equations [10,14] and often involves expensive calculations requiring parallel computing. Summaries of recent advances in the understanding of electroconvective instability in various applications are given by Chang et al. [15] and Nikonenko et al. [16,17,18].

All of the theoretical investigations of electroconvection consider systems under direct current (DC) electric fields and most often with ion-selective membranes as the ion-exchange interface. Reacting electrodes under DC fields are expected to result in similar electroconvective flows since their base state involves similar key physics; i.e., voltage-driven charge-selective transport of counterions towards the interface, while coions are blocked. Likewise, this combination leads to concentration polarization and formation of ESC layer hospitable to instability [19]. A class of inert electrode systems, however, offers a significantly different physics. While they drive transport in the bulk, their interface blocks both co- and counterions. Hydrodynamic instabilities near such inert electrode surfaces are mostly unexplored and not understood.

Davidson et al. [20] showed that a floating inert electrode in an electrolyte exposed to an external DC electric field, can induce electroconvection. In this case, counterions that migrate toward the surface are normally fluxed into the EDLs and subsequently migrated out by moving tangentially along the curved EDL. This makes the EDL effectively act as a charge-selective membrane discussed in previous studies. They reported similar electroconvective structures as those involved in membranes, but subject to a background electro-osmotic flow induced by the electrode itself. However, a simpler question, and a more practically relevant one, is what happens when inert electrodes themselves are the source of the driving voltage? When the driving voltage is DC, the answer is simple: no instability can be sustained in the long term, since the DC current of an inert electrode is zero, and thus there is no mechanism of injecting energy into the system. The question remains whether inert electrodes can sustain electroconvection when they are subject to alternating current (AC) electric fields.

Wide range of applications involve inert electrodes subject to AC voltage. Examples include dielectric barrier plasma discharges [21], electro-osmotic pumps [22,23], electrochemical impedance spectroscopy [24] and electrostatic comb drive actuators [25]. An external oscillatory time scale, imposed on top of intrinsic ones such as the charge-relaxation time and the resistor–capacitor (RC) charging time scale [26], complicates the mechanisms of hydrodynamic coupling with ion transport and the overall system level responses. Theories and extensive discussions of AC electro-osmosis in weakly and strongly nonlinear regimes can be found in the literature [24,27,28,29]. In particular, Olesen et al. [28] showed that the overall cell impedance and the critical voltage above which EDL demonstrates strongly nonlinear behavior are strong functions of the external AC frequency. Yet, the characterization of such systems in the context of electroconvective instability and mixing enhancement in higher spatial dimensions remains unexplored.

In this study we demonstrate existence and several regimes of electroconvection in AC voltage setups and near flat inert electrodes. A 2D aqueous binary electrolyte bounded by two flat inert electrodes is considered, and an external AC electric field at a single oscillation frequency is applied in the wall-normal direction. We show that at sufficiently high voltages (in units of thermal volts, defined in Section 2), the 1D solution becomes unstable in the direction tangential to the electrode surface, and electroconvective instability is triggered. This article investigates this new phenomenon over a wide range of external voltage magnitude, oscillation frequencies and ion diffusivity ratios, and assesses their impacts on the development of electroconvection.

This article is organized as follows: The computational setup is given in Section 2, followed by a description of a fully-conservative numerical scheme used to solve governing equations in Section 3. A detailed characterization of electroconvective instability is presented in Section 4 with emphasis on the effects of the amplitude and frequency of applied oscillatory voltage, followed by the effects of ionic diffusivity on electroconvection. Conclusions and suggested future works are discussed in Section 5.

## 2. Problem Setup

**Figure 1:**
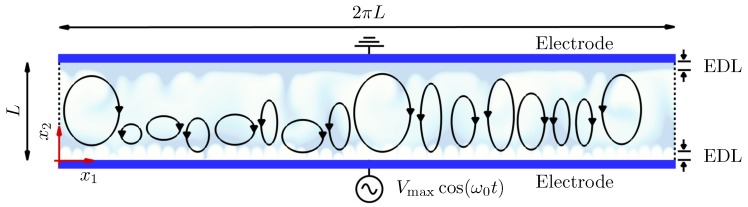
Computational domain. Also shown are schematics of large-scale vorticies and electric double layers (EDLs) (not to scale) near electrodes at $x_{2}=0$ and *L* (grounded). The domain is periodic in the $x_{1}$ direction.

We consider two parallel flat electrodes in a 2D domain subject to an AC voltage difference as shown in Figure 1. The fluid in between consists of a binary electrolyte with cations and anions of the same valency. The top and bottom walls of the fluid channel correspond to ideally-polarizable blocking electrodes with zero Faradaic current. Throughout this paper, $x_{1}$ and $x_{2}$ denote the horizontal and wall-normal directions, respectively. Within the channel, concentration of cations ($c^{+}$) and anions ($c^{-}$) having the same valency, ±z, is solved for. Superscripts + and − are used to denote quantities of cations and anions, respectively. Thermal equilibrium at room temperature is assumed, and ionic diffusivity for each species $D^{\pm }$ is uniform in space and constant in time. However, diffusivity is allowed to vary from one species to another. Transport equation for ionic species using the Nernst–Planck fluxes can be written as

(1) $$\frac{\partial c^{\pm }}{\partial t}+u\cdot \nabla c^{\pm }=\nabla \cdot \left(D^{\pm }\nabla c^{\pm }\right)\pm \nabla \cdot \left(D^{\pm }\frac{1}{V_{T}}c^{\pm }\nabla \phi \right),$$

where $u=\{u_{1},u_{2}\}$ is flow velocity, $V_{T}=k_{B}T/\left(ze\right)$ is the thermal volt with $k_{B}$, *T* and *e* being the Boltzmann constant ($1.38\times 10^{-23}$ J/K), absolute temperature and the elementary charge ($1.6\times 10^{-19}$ C), respectively, and ϕ is the electric potential. This is coupled with the Gauss’s law

(2) $$\nabla^{2}\phi =-\frac{ze(c^{+}-c^{-})}{\varepsilon },$$

where ε is the electrical permittivity.

Hydrodynamic transport is evaluated by directly solving the 2D Navier–Stokes equations in the incompressible, low-Reynolds limit. Thus, the nonlinear convection term is neglected. Instead, an electrohydrodynamic coupling term is included in the momentum equations.

(3) $$\begin{array}{c} \nabla \cdot u=0, \end{array}$$

(4) $$\begin{array}{c} \rho \frac{\partial u}{\partial t}=-\nabla p+\mu \nabla^{2}u-ze\left(c^{+}-c^{-}\right)\nabla \phi , \end{array}$$

where ρ is mass density, *p* is pressure and μ is dynamic viscosity. Due to the thermal equilibrium assumption, all thermodynamic quantities are uniform and constant.

Equations (1)–(4) are nondimensionalized by relevant reference quantities such as the channel height *L* (see Figure 1) for length, cation diffusivity $D^{+}$ for molecular diffusivity, cation diffusion time scale $L^{2}/D^{+}$ for time, diffusion velocity of cation $D^{+}/L$ for velocity, initial average salt concentration $c_{0}$ for ion concentration, the osmotic pressure $\mu D^{+}/L^{2}$ for pressure and $V_{T}$ for electric potential. As a result, six dimensionless parameters, as summarized in Table 1, characterize the electrohydrodynamics of the given system, namely, the electrohydrodynamic coupling constant $\kappa =\varepsilon V_{T}^{2}/\left(\mu D^{+}\right)$, nondimensional Debye screening length $\epsilon =\lambda_{D}/L$, Schmidt number $\mathrm{Sc}=\mu /(\rho D^{+})$, nondimensional maximum voltage $\Delta V=V_{\max}/V_{T}$, nondimensional oscillation frequency $\omega_{0}$ and the ratio of diffusivities $D^{-}/D^{+}$. The Debye screening length is defined by

(5) $$\lambda_{D}=\sqrt{\frac{\varepsilon k_{B}T}{2{\left(ze\right)}^{2}c_{0}}},$$

where $c_{0}$ is the mean concentration of both species averaged in the entire domain and is independent of time. In the analysis, it is useful to introduce normalized salt concentration $c=\frac{1}{2}\left(c^{+}+c^{-}\right)/c_{0}$ and charge density $\rho_{e}=\frac{1}{2}\left(c^{+}-c^{-}\right)/c_{0}$, which will be extensively used in Section 4.

Considering typical aqueous systems, κ=0.5 and Sc=1000 are used. The nondimensional Debye screening length is assumed to be $\epsilon =10^{-3}$ following Druzgalski et al. [8] and Davidson et al. [30]. While this ϵ is toward the larger limit of practical regimes, it is sufficiently small to reveal the essential physics without requiring significant computational cost. Our preliminary studies have shown that ΔV=180 is sufficient to develop electroconvective instability in the current setup, although smaller voltages also show similar, but less significant, instability. Following the 1D analysis of Olesen et al. [28], ΔV=180 corresponds to the strongly nonlinear regime where, depending on oscillation frequency, EDL loses quasiequilibrium nature due to severe salt depletion and admits the formation of ESC layer in their matched asymptotic analysis. Note that Olesen et al. [28] analyzed a half cell and thus their voltages are twice smaller than ours for an equivalent system (in other words, ΔV in the present study is equal to 2V of Olesen et al. [28]).

While ϵ, κ and Sc are kept fixed, the AC frequency and diffusivity ratio are systematically varied to investigate the onset of electroconvective instability. The oscillation frequency is scaled by the RC frequency of the equivalent circuit model, $\omega_{\mathrm{RC}}=2/\left(RC\right)$, where *R* and *C* are bulk resistivity based on mean concentration and linear capacitance of the nominal Debye layer, respectively. For example, if cation and anion diffusivities are the same ($D^{-}/D^{+}=1$), the dimensionless RC frequency is given by 2/ϵ. Based on this, it is convenient to introduce a reduced frequency $\tilde{\omega }=\omega /\omega_{\mathrm{RC}}$, where $\tilde{\omega }=1$ corresponds to the nondimensional characteristic RC frequency. Thus, the reduced frequency for the external AC field is written as ${\tilde{\omega }}_{0}$.

For cases in which we study the effects of diffusivity ratio, $D^{-}/D^{+}$, cation diffusivity is used as a reference, and anion diffusivity is varied. For a fixed voltage ΔV=180, three different diffusivity ratios are considered, namely $D^{-}/D^{+}=1,5$ and 10. Several effective diffusivities, $D_{\mathrm{eff}}$, can be defined using ionic diffusivities. If the diffusivity ratio is not equal to unity, electroneutrality in the bulk of a binary electrolyte requires that the salt diffuses at $D_{h}=\frac{2D^{+}D^{-}}{D^{+}+D^{-}}$, which is a harmonic mean of two diffusivities. However, for the two parallel flat electrode setup, the characteristic RC frequency is associated with the arithmetic mean $D_{m}=\frac{1}{2}\left(D^{+}+D^{-}\right)$. Using the effective diffusivity, an effective diffusion velocity $u_{\mathrm{diff}}=D_{\mathrm{eff}}/L$ can be defined. Similarly, an effective diffusion time scale can be defined as $\tau_{\mathrm{diff}}=L^{2}/D_{\mathrm{eff}}=L/u_{\mathrm{diff}}$.

The simulation domain is periodic in the $x_{1}$ direction with length 2πL, as shown in Figure 1. In similar electroconvective flows under DC voltages, Druzgalski et al. [8] showed that autocorrelation of velocity fluctuations remains close to zero for separations greater than 2L in the periodic direction and thus the artifacts of using the periodic condition is insignificant. As pointed out by Druzgalski and Mani [12], the current aspect ratio 2π appears sufficiently high, given much stronger external voltages and chaotic hydrodynamic responses of the current electrolyte setup. On the electrode surfaces at $x_{2}/L=0$ and 1, the no-slip condition is used for fluid velocity, and ion fluxes normal to the surfaces vanish due to zero electrochemical reaction. For electric potential, the top wall is grounded, and the bottom wall has an externally imposed sinusoidal voltage $V_{\max}\cos\left(\omega_{0}t\right)$ at a single constant (angular) frequency $\omega_{0}$. The angle θ is used to describe the phase of the AC voltage oscillation; for example, θ=0 corresponds to the bottom electrode having the maximum positive voltage $V_{\max}$, while θ=π corresponds to $\phi \left(x_{2}/L=0\right)=-V_{\max}$.

At t=0, ionic concentrations are unity for both ionic species, and fluid velocities are set to be zero everywhere. Initial random disturbances are imposed on $c^{\pm }$ with the maximum perturbation being $5\times 10^{-12}c_{0}$. Preliminary studies have shown that the perturbation magnitude does not have a meaningful influence on the development of electroconvective instability after the first few oscillation periods for which initial transient effects are still non-negligible.

At sufficiently large voltages, the classical Poisson–Boltzmann model (also used in this study) predicts very high concentration of counterions near charged interfaces, even in dilute-solution limits. The Boltzmann statistics assumes pointwise ionic species and allows counterions to be congregated at unphysically high concentration. In reality, volume constraints of finite-size ions and other mechanisms inhibit such high concentration of ions. This crowding of ions can to some degree be alleviated by including a compact Stern layer near the interfaces, which provides additional surface capacitance so that the excess potential across the Debye layer can be reduced at moderate applied voltages. An alternative (and presumably more realistic) approach is to employ the modified Poisson–Nernst–Planck (MPNP) model [31,32] so that ion crowding within very thin layers can be effectively limited by steric effects. Extensive comparisons and discussions in AC electrode setups are given by Olesen et al. [28]. Consideration of these realistic effects requires to include additional modeling parameters such as surface capacitance for a Stern layer and maximum volume fraction for steric constraints, adding two more dimensions to the present parameter space. Given that there is no strong consensus on the values of those additional parameters, their effects should be assessed comprehensively for a range of values. In this investigation, we avoid such a comprehensive study, and ignore both steric and Stern layer effects, to allow a focus on controllable parameters such as the applied frequency and voltage.

In experimental setups involving aqueous electrolytes, applying sufficiently high AC voltages at sufficiently low frequencies can lead to electrolysis of water near the surface and generation of bubbles, which would bring additional complexities such as two-phase mass transfer and contact-line dynamics. These complexities need to be addressed in a more advanced version of the present study. In this study, our goal is to develop an understanding of electrokinetic effects by isolating them from other complexities. Therefore, our work is relevant to systems with highly stable surfaces and chemistries where the onset of bubble generation is postponed beyond the considered frequency–voltage regimes.

## 3. Numerical Methods

The governing equations, Equations (1)–(4), are solved (in their nondimensionalized forms) using the numerical scheme proposed by Karatay et al. [14] with the modification of Davidson [33] to enforce discrete conservation at all iteration levels. The simulation domain is decomposed into a number of Cartesian control volumes, and variables are staggered so that velocities and ion fluxes are defined at face centers, while the other quantities are located at cell centers. The governing equations are integrated over control volumes and discretized with second-order accuracy in space. For numerical stability, simple averaging is used to compute face-centered quantities, instead of accounting for nonuniform grid spacing.

The presence of very thin EDLs near electrode surfaces renders the system of discretized equations numerically stiff, requiring a prohibitively small computational time-step size. This difficulty is alleviated by using a semi-implicit treatment of fluxes in the wall-normal direction. In the periodic transverse direction, fluxes are computed explicitly in time, but Fourier transforms are used to time advance the momentum equations and obtain solutions of the Poisson equations to take advantage of transverse decoupling and reducing the bandwidth of matrices associated with implicit time advancement. The second-order backward Euler method is used for temporal discretization. At each time step, nonlinear terms are linearized, and the system of linearized governing equations is iteratively solved until convergence of the full-nonlinear implicit system is achieved.

The computational grid in the periodic $x_{1}$ direction is uniform in spacing. In the wall-normal $x_{2}$ direction, a hyperbolic tangent profile is used to create nonuniformly spaced grid points clustered near the electrodes. Grid spacings in the $x_{2}$ direction are symmetric with respect to $x_{2}/L=0.5$ (the channel centerline). In the $x_{1}$ and $x_{2}$ directions, respectively, 1024 and 200 control volumes are used. Uniform grid spacing $\Delta x_{1}$ corresponds to $6\lambda_{D}$. In the $x_{2}$ direction, approximately 13 cells are used to resolve the EDL, and the minimum and maximum grid spacings are $\Delta x_{2,\min}/\lambda_{D}=0.05$ and $\Delta x_{2,\max}/L=0.018$, respectively.

Computational time-step size is kept constant as $\Delta t/\tau_{\mathrm{diff}}=10^{-7}$. This corresponds to $10^{4}$ time steps per oscillation period at the highest oscillation frequency considered in this study, ${\tilde{\omega }}_{0}=\pi$. The number of iterations per time step ranges from 2 to 10, primarily depending on applied voltage and diffusivity ratio. An $L_{\infty }$-error tolerance is prescribed to be $10^{-3}$ for convergence. Simulations are time advanced for 10 oscillation periods, after which statistics are collected for another 10 periods.

The simulation code has been verified by using the manufactured solution method [33]. Also, a series of comparisons are made against 1D asymptotic solutions of a binary symmetric mixture in contact with a cation-selective membrane under applied DC voltages. Ion concentration, charge density and voltage–current curve show good agreement with the corresponding analytic solutions and previous studies.

## 4. Results

### 4.1. Electroconvective Instability of Binary Symmetric Electrolytes ($D^{-}/D^{+}=1$)

#### 4.1.1. Evolution of Electrohydrodynamic Structures Under External AC Voltages

**Figure 2:**
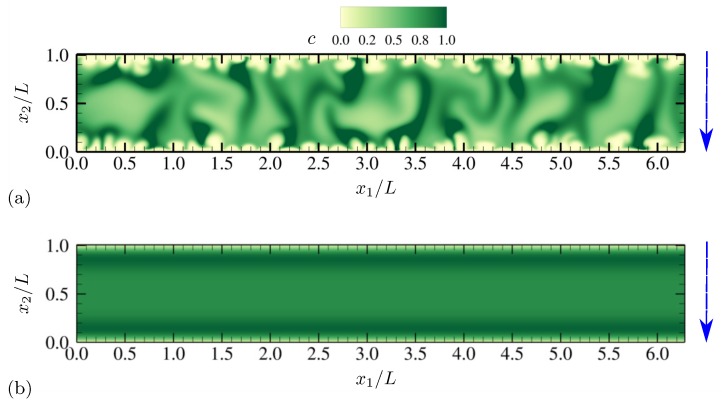
Instantaneous contours of salt concentration *c* at θ=π for (**a**) ΔV=180 and (**b**) ΔV=40. The oscillation frequency is ${\tilde{\omega }}_{0}=0.063$ for both cases. The vertical arrows denote the direction of the applied electric field. The diffusivity ratio is $D^{-}/D^{+}=1$.

Electroconvective instability induced by external DC electric fields demonstrates distinct qualitative features represented by an array of counter-rotating vortices near ion-selective membranes [8,10,20]. Similar observation can be made for the present setup where external AC voltage is applied. Figure 2a shows the instantaneous contours of salt concentration *c* at the phase when the lower electrode has the minimum voltage $-V_{\max}$. The AC frequency is ${\tilde{\omega }}_{0}=0.063$, which is well below the RC frequency of the present electrokinetic system and a frequency where optimal hydrodynamic responses are reported in Section 4.1.4. The near-electrode regions of severe ion depletion (bright color) have thickness comparable to 20% of the channel height. The bulk solution is strongly nonuniform in its salt distribution, and its salt concentration is far from unity. Also, characteristic length scales of structures range up to scales comparable to the channel height *L*.

With all the conditions described in Section 2 the same, the maximum voltage is lowered to ΔV=40. Figure 2b shows the instantaneous contours of *c* at the same phase as the one in Figure 2a. Ion distribution is essentially 1D varying in the $x_{2}$ direction, and there is no sign of electroconvection nor transverse instability. Salt concentration stays nearly unity in the bulk solution. This shows that electroconvective instability begins to develop above a certain voltage, similar to the cases where DC electric fields are applied to electrolytes in contact with ion-selective membranes [8].

**Figure 3:**
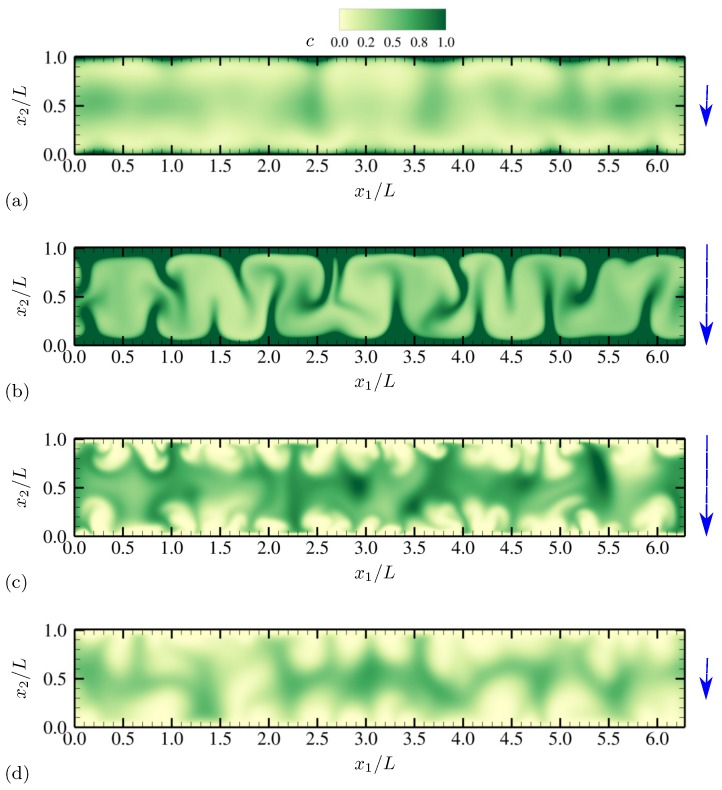
Time series of salt concentration *c* for half an oscillation period at (**a**) θ=0.64π, (**b**) 0.88π, (**c**) 1.12π and (**d**) 1.4π. The vertical arrows denote the direction and relative magnitude (not to scale) of the applied electric field. The diffusivity ratio is $D^{-}/D^{+}=1$.

**Figure 4:**
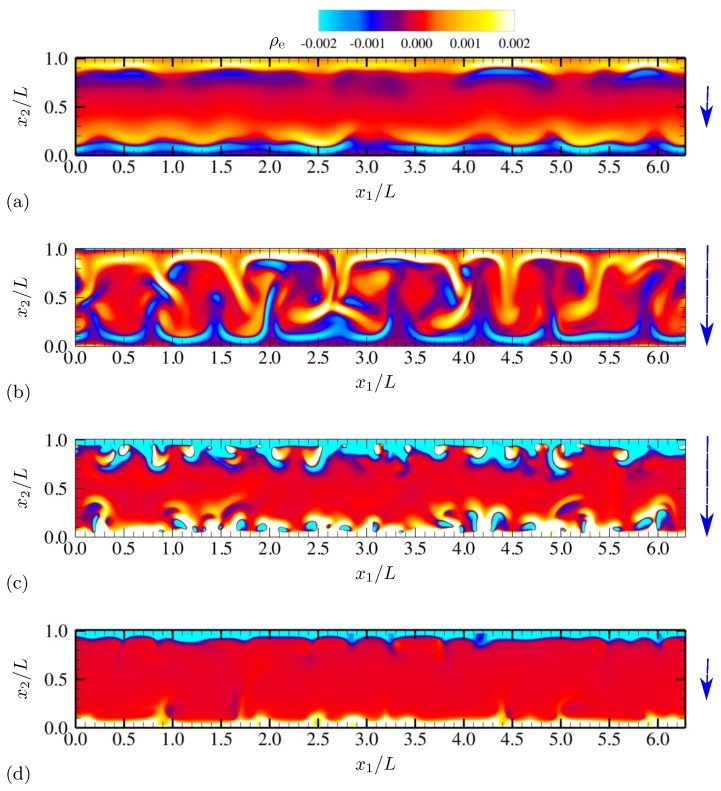
Time series of charge density $\rho_{e}$ for half an oscillation period at (**a**) θ=0.64π, (**b**) 0.88π, (**c**) 1.12π and (**d**) 1.4π. The vertical arrows denote the direction and relative magnitude (not to scale) of the applied electric field. The diffusivity ratio is $D^{-}/D^{+}=1$.

**Figure 5:**
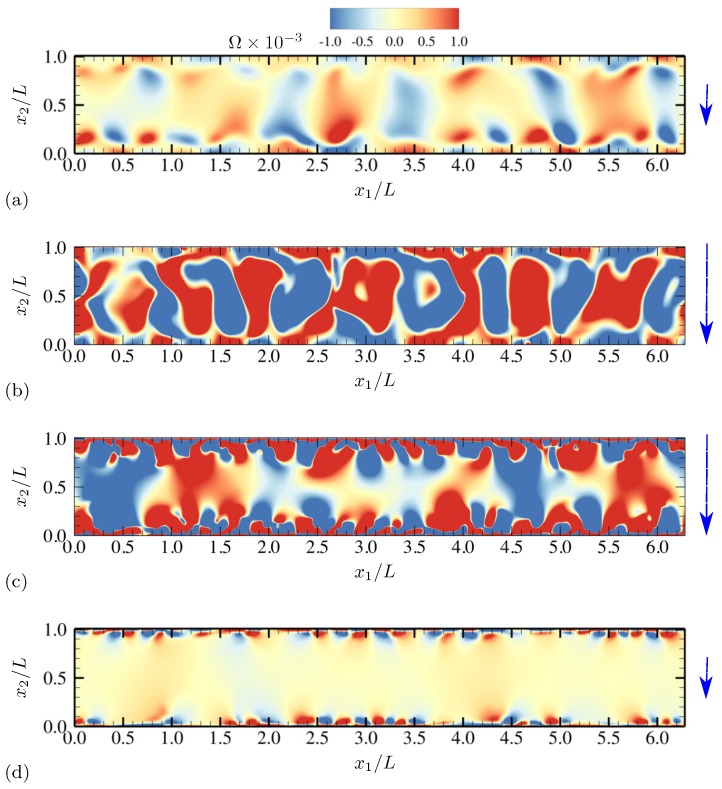
Time series of vorticity Ω for half an oscillation period at (**a**) θ=0.64π, (**b**) 0.88π, (**c**) 1.12π and (**d**) 1.4π. The vertical arrows denote the direction and relative magnitude (not to scale) of the applied electric field. The diffusivity ratio is $D^{-}/D^{+}=1$.

More detailed qualitative features of electroconvective instability can be observed by plotting salt concentration at each phase of AC voltage oscillation. In Figure 3, snapshots corresponding to a half oscillation period are shown (corresponding to phase angles between π/2 and 3π/2). Thus, the electrode at $x_{2}=0$ stays negatively charged (the upper electrode is grounded). The applied voltage at the lower electrode takes its minimum $-V_{\max}$ between Figure 3b,c. Symmetry in geometry and diffusivity ensures that the results are similar during the other half oscillation period. Similar plots are obtained for charge density and vorticity $\Omega =\frac{\partial u_{2}}{\partial x_{1}}-\frac{\partial u_{1}}{\partial x_{2}}$ (scaled by $u_{\mathrm{diff}}/L$) at the same phases in Figure 4 and Figure 5, respectively.

As instantaneous voltage difference across the channel increases (between Figure 3a,b, and Figure 4a,b), double layers on both electrodes are charged, and more coions are expelled from electrodes, while more counterions are attracted towards the electrodes. At the maximum voltage difference (between Figure 3b,c, and Figure 4b,c), near-electrode structures undergo drastic qualitative changes. In Figure 3c and Figure 4c, similarities to Druzgalski and Mani [12] are observed, namely strong ion depletion correlated with high charge density. Such severe ion depletion leads to the formation of ESC and transversely organized structures. As the voltage drop returns close to zero in Figure 3d and Figure 4d, the organized structures are relaxed primarily via diffusion.

A notable difference with Druzgalski and Mani [12] is observed at the phase when the double layer is charging (in Figure 3b or Figure 4b). As the voltage drop becomes stronger, a layer of highly concentrated ions, but with coionic charge, is formed near each electrode. This layer is then advectively stretched (see Figure 5b) via thin normal fingers spaced in the $x_{1}$ direction with characteristic length scales of O(L). In other words, during this phase each electrode simultaneously acts as a “membrane” and a “reservoir” for the opposite electrode, analogous to the observations made in Druzgalski and Mani [12].

Discussions so far concern phenomenological descriptions of electroconvection caused by external AC electric fields. Though qualitative, they demonstrate the importance of critical voltage at which electroconvection emerges and rich transient dynamics depending on oscillation phase when electroconvection is pronounced. Videos S1–S3 showing the time-resolved dynamics of AC electroconvective instability for a single oscillation period can be found in Supplementary Materials. In the next section, quantitative descriptions of electroconvective instability are provided.

#### 4.1.2. Statistics

**Figure 6:**
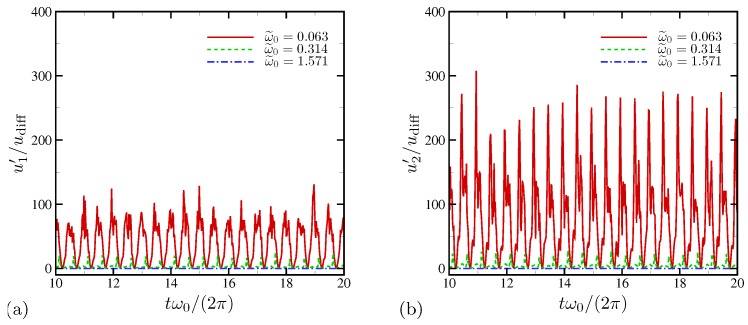
Instantaneous fluctuations of (**a**) horizontal and (**b**) wall-normal velocities. Measurement is made at the channel centerline, and RMS fluctuations are computed with respect to the mean in the horizontal direction.

The instantaneous fluctuating horizontal velocity (with respect to the mean in the horizontal direction) is shown in Figure 6a for several oscillations frequencies. Strong intermittent fluctuations are observed at ${\tilde{\omega }}_{0}=0.063$ where electroconvection is reported. Wall-normal velocity fluctuations are two to three times higher than horizontal velocity fluctuations, as can be seen in Figure 6b.

**Figure 7:**
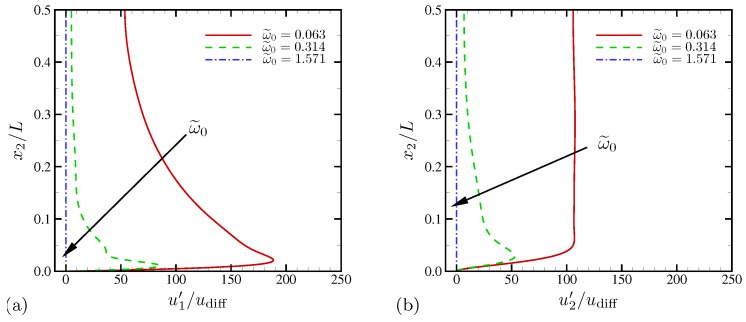
RMS velocity fluctuations versus wall normal coordinate for (**a**) horizontal and (**b**) wall-normal velocities.

The root-mean-square (RMS) profiles of horizontal velocity fluctuations with respect to time and the horizontal direction are shown as a function of wall-normal distance in Figure 7a. Strong peaks are observed very near the electrode surface except for ${\tilde{\omega }}_{0}=1.571$ where the system is essentially 1D. At ${\tilde{\omega }}_{0}=0.063$, $u_{1}^{\prime }$ does not decay to a negligible value at the centerline anymore, consistent to Figure 6a. Figure 7b shows the profiles of RMS wall-normal velocity. The strong electroconvective instability at ${\tilde{\omega }}_{0}=0.063$ creates substantially higher and nearly constant level of wall-normal velocity fluctuations across 90% of the channel width. Unlike the higher frequency cases, $u_{2}^{\prime }$ does not decay toward the channel centerline and rather takes its maximum value there, which confirms that there is a global hydrodynamic coupling across the channel.

**Figure 8:**
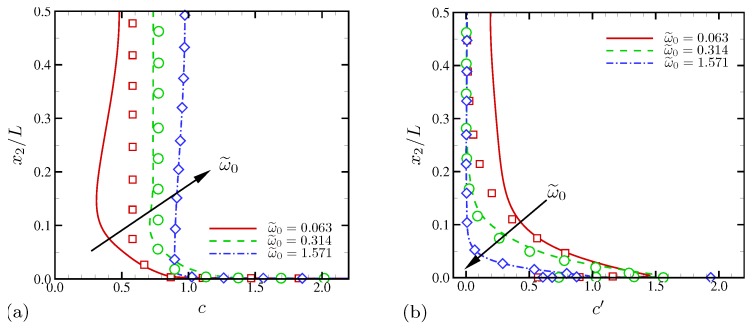
The wall-normal profiles of (**a**) time-averaged salt concentration and (**b**) salt fluctuation RMS. Symbols indicate the solutions from the corresponding 1D simulations.

The time-averaged profiles of salt concentration are shown in Figure 8a for the three different oscillation frequencies, respectively. Statistics are also averaged in the periodic $x_{1}$ direction. Also shown in Figure 8a as symbols are the average salt profiles for the corresponding 1D simulations using the same numerical method. At ${\tilde{\omega }}_{0}=0.063$, the minimum average salt drops to 0.3 at $x_{2}/L\approx 0.1$. The bulk salt concentration is lower than 0.5, consistent to the qualitative observations made in Figure 3. Comparison with the corresponding 1D solution shows that the inner structure near the electrode ($x_{2}/L≲0.03$) remains 1D on average. However, electroconvective instability causes significantly higher degree of ion depletion all across the channel, and the outer-layer ionic distribution is not well predicted by the 1D model. At higher frequencies, variations of average salt are nearly indistinguishable from those of the corresponding 1D solutions. The RMS profiles of salt fluctuation in Figure 8b provides clear distinction between strong and mild electroconvective instability. Near the electrode surface ($x_{2}/L≲0.05$), fluctuation levels are relatively high for all frequencies, regardless of whether there is electroconvection. However, strong electroconvective instability at ${\tilde{\omega }}_{0}=0.063$ maintains salt fluctuations higher than 40% of its bulk concentration across the channel, while those at higher frequencies decay to zero at the centerline. The corresponding 1D solution at ${\tilde{\omega }}_{0}=0.063$ shows a similar decay of fluctuations toward the centerline. The results in Figure 8 show that electroconvective instability induced by external AC electric field is a strictly multi-dimensional phenomenon (in particular, outside the double layer) and cannot be accurately described using a 1D Poisson–Nernst–Planck model. However, the inner-layer structures ($x_{2}/L≲0.03$) are still close to those of the 1D analysis.

**Figure 9:**
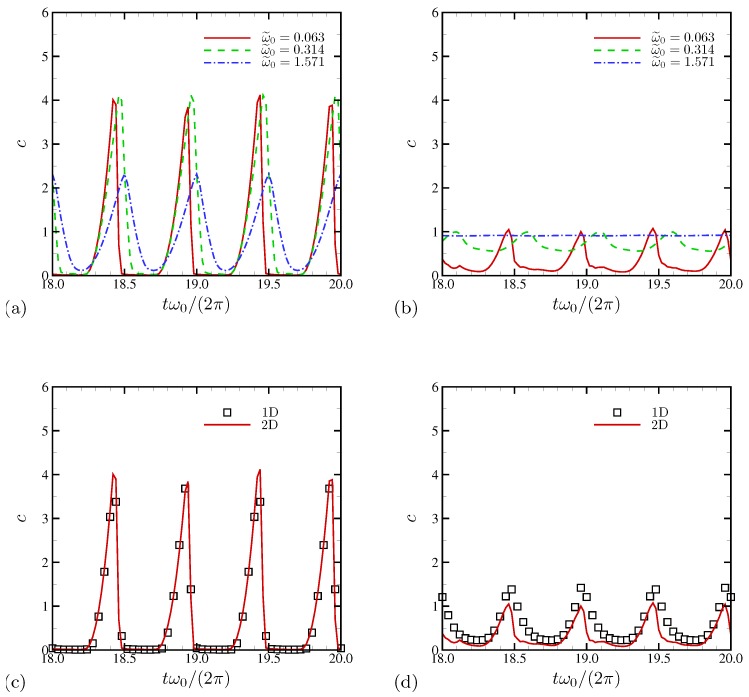
Time history of instantaneous salt concentration averaged in the horizontal direction at (**a**) $x_{2}/L=0.01$ and (**b**) $x_{2}/L=0.1$. In (**c**,**d**), only the results for ${\tilde{\omega }}_{0}=0.063$ are shown for comparison with the corresponding 1D simulation. Two oscillations periods are plotted.

In Figure 9a,b, instantaneous salt concentration averaged in the periodic $x_{1}$ direction is shown at $x_{2}/L=0.01$ and 0.1, respectively. Only two oscillation periods are shown for clarity. At ${\tilde{\omega }}_{0}=0.063$ where electroconvection is pronounced, severe salt depletion is observed in the ESC region lasting more than 50% of each oscillation period (see Figure 9a). Away from the electrode at $x_{2}/L=0.1$ (Figure 9b), salt concentrations for higher AC frequencies more or less relax to the value of the bulk electrolyte $c_{0}=1$, while the minimum concentration for ${\tilde{\omega }}_{0}=0.063$ is only 20% of the bulk concentration.

We revisit the earlier observation made in Figure 8a regarding agreement between 1D and 2D mean salt concentrations near the electrode surface. In Figure 9c,d we show that this agreement persists even at instantaneous levels. At $x_{2}/L=0.01$, both 1D and 2D predictions are indistinguishable, consistent with what was observed in Figure 8a. However, ion concentrations at $x_{2}/L=0.1$ are consistently lower for 2D simulation, as shown in Figure 9d. At higher AC frequencies (with no electroconvection), comparisons at both $x_{2}$ locations give nearly identical results between 1D and 2D simulations (not shown here).

#### 4.1.3. Spectral Analysis

**Figure 10:**
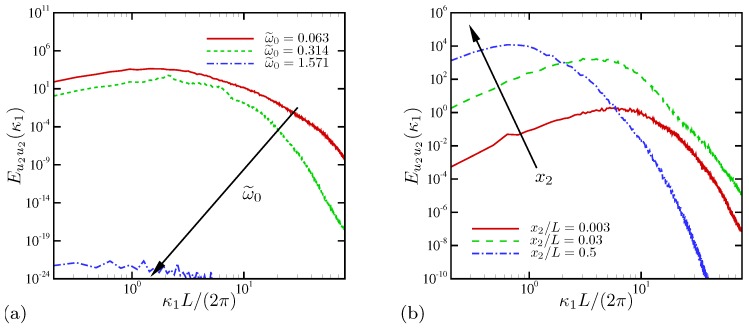
Spatial power spectral density of wall-normal velocity at (**a**) $x_{2}/L=0.1$ for three oscillation frequencies and (**b**) several positions from the electrode surface for ${\tilde{\omega }}_{0}=0.063$.

Figure 10a shows the spatial power spectral density (PSD) of wall-normal velocity for three different frequencies. The nominally 1D case at ${\tilde{\omega }}_{0}=1.571$ has negligibly small spectral energy, presumably the residue of the initial random perturbations. At lower frequencies, electroconvective instability develops a broadband distribution of spectral energy similar to energy spectra reported by Druzgalski and Mani [12] for applied DC electric fields. In Figure 10b, the variation of spectral energy at several wall-normal positions is shown for ${\tilde{\omega }}_{0}=0.063$. Slightly outside the nominal EDL ($x_{2}/L=0.003$), spectral energy takes its maximum value at $\ell_{1}=2\pi /\kappa_{1}\approx L/8$. At $x_{2}/L=0.03$ approximately outside the ESC layer [28], a significant amplification of energy is found at all wavenumbers. Also, the spectral peak is shifted toward larger scales to $\ell_{1}\approx L/4$. At the channel centerline $x_{2}/L=0.5$, the peak is further shifted to $\ell_{1}\approx 1.5L$. Thus, the most energetic fluid motion in the horizontal direction at the centerline is 50% larger than the channel width *L*. Druzgalski and Mani [12] also reported peaks approximately at 1.5L in their kinetic energy spectra away from the ion-selective membrane when electroconvection takes place under strong external DC voltages. Another consistent observation is that energetic structures become progressively smaller closer to the interface, as found in traditional turbulent flows [13].

**Figure 11:**
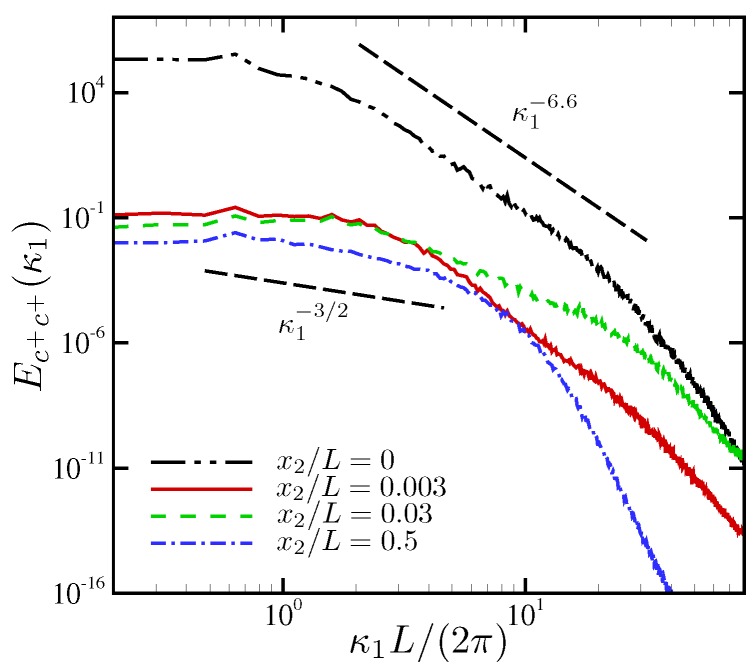
Spatial power spectral density of cation concentration for ${\tilde{\omega }}_{0}=0.063$.

Similar analysis can be done for ionic concentration for ${\tilde{\omega }}_{0}=0.063$. In Figure 11, the PSD of cation concentration is plotted at several wall-normal locations, including the first-cell location denoted by $x_{2}/L=0$. A spectral energy decay at $\kappa_{1}^{-3/2}$ is observed at an intermediate wavenumber range, a rate also observed by Druzgalski and Mani [12] for external DC voltages. The spectral energy of ionic fluctuations stay more or less the same at $\ell_{1}≳0.2L$, while at wavenumbers larger than $\kappa_{1}L/\left(2\pi \right)\approx 5$, energy contained at small scales shows variations by several order of magnitudes. Very near the electrode, a distinct power-law decay of spectral energy at $\kappa_{1}^{-6.6}$ is observed for almost two decades of wavenumber.

**Figure 12:**
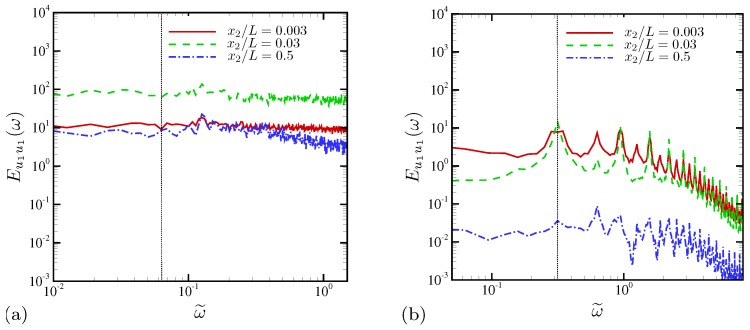
Temporal power spectral density of horizontal velocity at several wall-normal locations. The vertical dotted lines indicate the external AC frequencies (**a**) ${\tilde{\omega }}_{0}=0.063$ and (**b**) 0.314, respectively.

Temporal PSD is computed at a number of wall-normal locations and averaged in the periodic $x_{1}$ direction. Figure 12 shows temporal PSD of the horizontal velocity $u_{1}$ at several wall-normal locations. Strong electroconvective instability at ${\tilde{\omega }}_{0}=0.063$ (Figure 12a) results in drastically different temporal dynamics compared with mild electroconvective instability at ${\tilde{\omega }}_{0}=0.314$ (Figure 12b). In Figure 12a, outside the double layer ($x_{2}/L\geq 0.003$), temporal fluctuations are strong, and fluctuation energy is almost equally distributed over frequencies more than two decades up to $\tilde{\omega }=2$. This is also the case at the channel centerline. Mild peaks are observed at $\tilde{\omega }=0.126$, corresponding to a harmonics of the fundamental AC frequency due to the periodic attraction and repellence of ionic species occurring twice per oscillation period [28]. Slightly outside the ESC layer ($x_{2}/L=0.03$), temporal fluctuation energy takes the maximum values over all frequencies. When electroconvective instability is not significantly amplified and confined very near the electrode (Figure 12b), individual frequency peaks associated with the AC oscillation frequency appear as well as their higher harmonics. Compared with Figure 12a, it is clear that nonlinearity necessary to induce global electroconvective instability does not sufficiently develop at this higher oscillation frequency ${\tilde{\omega }}_{0}=0.314$.

**Figure 13:**
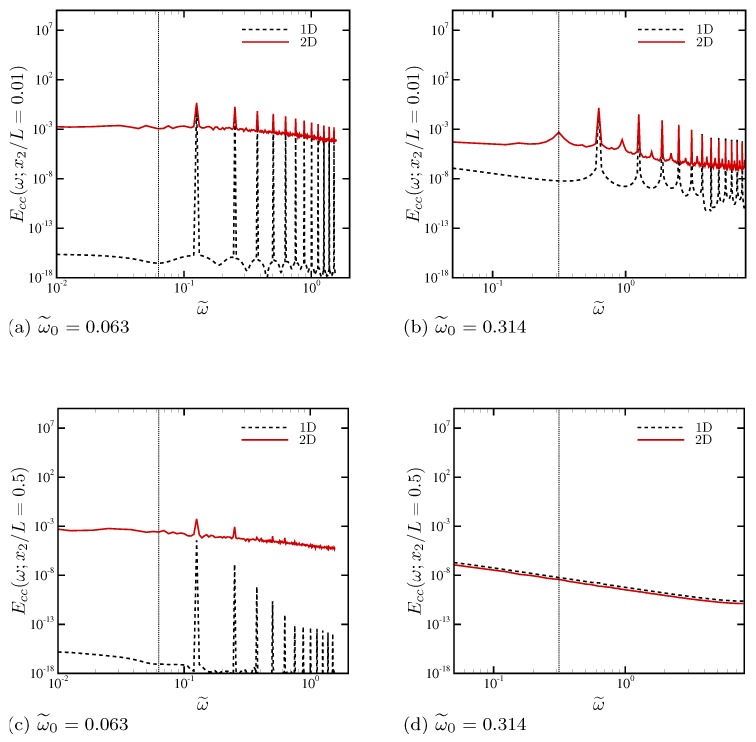
Comparison of temporal power spectral density of salt concentration at $x_{2}/L=0.01$ for (**a**) ${\tilde{\omega }}_{0}=0.063$ and (**b**) ${\tilde{\omega }}_{0}=0.314$, and at $x_{2}/L=0.5$ for (**c**) ${\tilde{\omega }}_{0}=0.063$ and (**d**) ${\tilde{\omega }}_{0}=0.314$.

In Figure 13, the temporal PSDs of salt concentration are compared with those of the corresponding 1D simulations. Near electrodes (Figure 13a,b), the frequencies corresponding to twice the external AC frequencies $\omega_{0}=0.063$ and 0.314 take a maximum, respectively. Their first and higher harmonics, namely $2n{\tilde{\omega }}_{0}$ where n=1,2,⋯, become predominant relative to the other frequencies. For ${\tilde{\omega }}_{0}=0.063$ which involves a strong electroconvective instability (Figure 13a), temporal PSD is substantially amplified across all frequency ranges. At a higher AC frequency ${\tilde{\omega }}_{0}=0.314$ where electroconvective instability is not significant (Figure 13b), difference between the 1D and 2D simulations is much smaller. Such drastic amplification in PSD over all frequency ranges is indicative of increased chaotic behavior in the system. At the channel centerline (Figure 13c,d), two different AC frequencies result in substantially different temporal PSD. With strong electroconvective instability (Figure 13c), electrolytes exhibit strong temporal fluctuations at the centerline as well. However, at a higher frequency ${\tilde{\omega }}_{0}=0.314$ (Figure 13d), the bulk electrolyte returns to essentially 1D solution without any discernible frequency peaks.

#### 4.1.4. Electric Current and System-Level Responses

**Figure 14:**
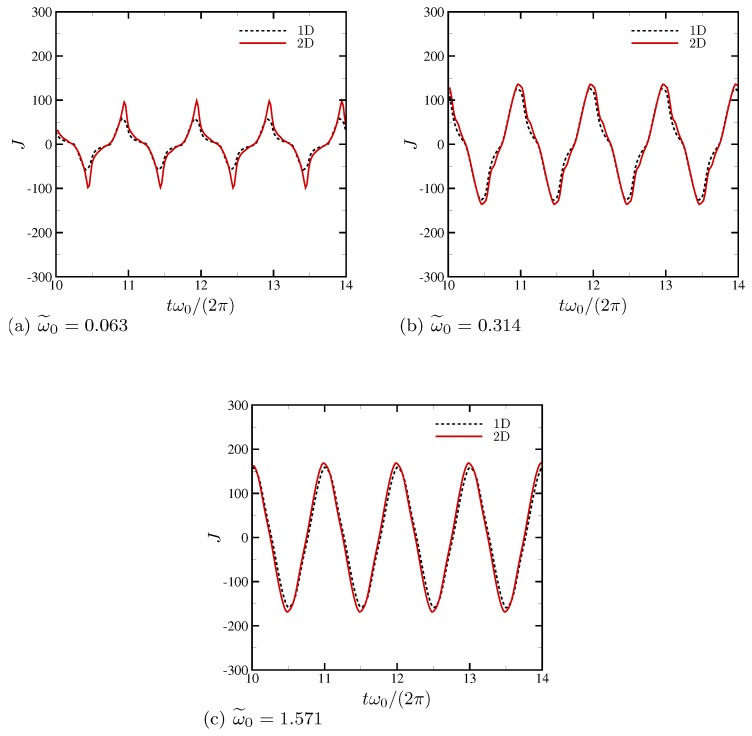
Surface current per unit area for (**a**) ${\tilde{\omega }}_{0}=0.063$, (**b**) 0.314 and (**c**) 1.571. Four oscillations periods are plotted.

Surface current density at the electrode surface is compared with that of the corresponding 1D simulations in Figure 14. Assuming a perfect conductor, current per unit area *J* is calculated by first computing surface charge per unit area $q^{\prime \prime }=-\varepsilon \nabla$ϕ·n, where n is the surface normal vector pointing toward the electrolyte. Then, $q^{\prime \prime }$ is numerically differentiated in time to obtain *J*. At ${\tilde{\omega }}_{0}=1.571$ where the response of the electrolyte is essentially 1D and electrohydrodynamic coupling is negligible, current is nearly sinusoidal and closely follows that of the corresponding 1D simulation. However, the presence of electroconvective instability at ${\tilde{\omega }}_{0}=0.063$ increases the maximum *J* by a factor of two compared with the corresponding 1D setup, as can be seen in Figure 14a. The wall-normal component of current density computed at the channel centerline is nearly identical to those computed at the electrode surface.

**Figure 15:**
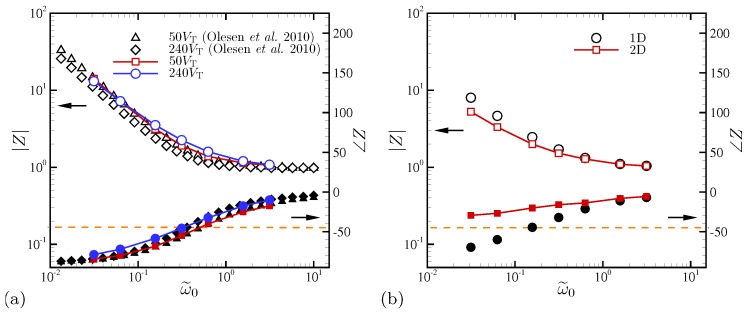
(**a**) The Bode plot of the overall cell impedance *Z* for the strictly 1D setup. To match the conditions of Olesen et al. [28], the EDL thickness is adjusted to be $\epsilon =5\times 10^{-4}$ and the applied maximum AC voltages are ΔV=50 and 240. (**b**) The overall cell impedance for the nominal 2D setup. The horizontal dashed line corresponds to $∠Z=-45^{\circ }$.

Following Olesen et al. [28], the overall cell impedance (or the generalized impedance) versus the AC oscillation frequency ${\tilde{\omega }}_{0}$ is calculated to characterize the system-level response. For comparison with Olesen et al. [28], a series of 1D simulations are conducted at $\epsilon =5\times 10^{-4}$ and at applied AC voltages of ΔV=50 and 240. It should be noted that Olesen et al. [28] uses the half-channel height as a reference length and their maximum voltage drop 2V is equal to ΔV in the present study. Additionally, in Olesen et al. [28] a finite size Stern layer thickness (δ=0.3 in their notation) is used. However, in our reproduction of the 1D simulations, to be consistent with our 2D simulations, we do not include a Stern layer. Nevertheless, this has minimally changed the plots, as will be seen in Figure 15.

Figure 15 compares the magnitude and phase angle of the overall cell impedance at applied voltages of ΔV=50 and 240, respectively. At ΔV=50, both 1D and 2D solutions show good agreement in magnitude and phase over all frequencies. However, at ΔV=240, some mild discrepancies are observed at intermediate frequencies.

Figure 15b shows the overall cell impedance computed for our nominal setup ($\epsilon =10^{-3}$ and ΔV=180). Both 1D and 2D simulation results are plotted. As discussed by Olesen et al. [28], the system response is nearly ohmic at frequencies higher than the RC frequency (or equivalently, ${\tilde{\omega }}_{0}\geq 1$), and the 1D and 2D results are indistinguishable. However, as the oscillation frequency is reduced, the system responses deviate from simple capacitor-like behaviors observed in the 1D setup. Instead, the system is characterized by reduced impedance amplitude (at most, by a factor 1.5) and reduced phase difference between the applied voltage and current. Again, this is attributed to electroconvective instability enhancing the convective transport and mixing of ionic species across the channel, and thus reducing the effective resistance of the electrolytes.

Olesen et al. [28] introduces the characteristic frequency at which contributions from resistive and capacitative components of the overall cell impedance switch. Specifically, this corresponds to a frequency at the phase angle of the impedance $∠Z=-45^{\circ }$ denoted by a horizontal dashed line in Figure 15. A notable difference between the 1D and 2D simulations is that as ${\tilde{\omega }}_{0}$ is reduced and electroconvective instability is pronounced, the characteristic frequency is significantly reduced which is consistent with increased effective current in the same figure.

**Figure 16:**
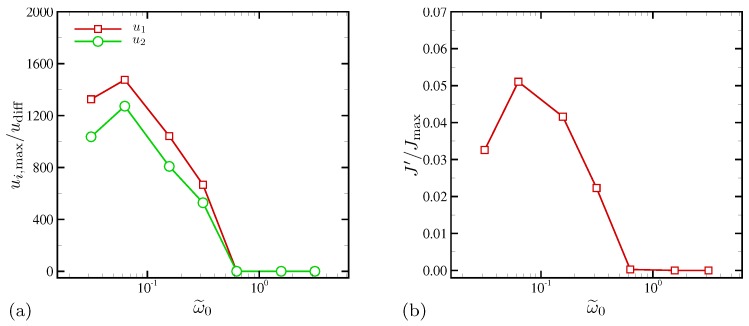
(**a**) Normalized maximum velocities and (**b**) normalized current density fluctuations in the transverse direction. In (**b**), $J_{\max}$ is obtained by taking the maximum of surface current density averaged in the $x_{1}$ direction.

The degree of electroconvective motions induced by the applied external AC voltage can be summarized by plotting the maximum values of velocity magnitudes as shown in Figure 16a. At ${\tilde{\omega }}_{0}\geq 0.628$, maximum velocities are negligibly small, indicating that the electrolytes behave essentially like a 1D system. As seen previously, hydrodynamic responses are strong at ${\tilde{\omega }}_{0}=0.063$. As ${\tilde{\omega }}_{0}$ becomes smaller than 0.063, induced maximum velocity magnitudes become smaller. This is expected since in the limit of zero frequency (DC limit) there must be no instability due to lack of mechanism for injecting energy into the electrolytes bounded by inert blocking electrodes. What is surprising is that the maximum electroconvection occurs at frequencies much smaller than the RC frequency on the order of ${\tilde{\omega }}_{0}=O\left(10^{-2}\right)$. Different measures based on velocity can be devised to characterize the responses, although no further efforts are made in this study.

Similar trends are obtained by evaluating normalized current density fluctuations in the $x_{1}$ direction, as shown in Figure 16b. The surface current density *J* is subtracted by its instantaneous mean in the $x_{1}$ direction, and its instantaneous RMS fluctuations are computed and time averaged. Thus, it characterizes the level of current fluctuations in the horizontal direction.The maximum current density RMS (≈5% of the maximum current density) occurs at ${\tilde{\omega }}_{0}=0.063$, the same as the peak frequency in Figure 16a. However, the peak in Figure 16b is defined better and presumably more robust since the maximum velocities used in Figure 16a are prone to intermittency in velocity fields as applied voltages increase further.

**Figure 17:**
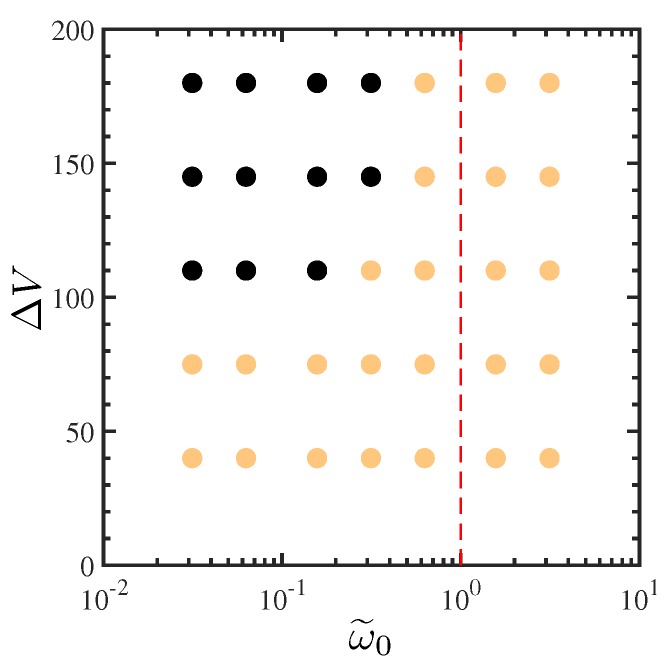
A frequency–voltage diagram for AC electroconvection of a binary symmetric electrolyte ($D^{-}/D^{+}=1$). Each symbol corresponds to a different pair of oscillation frequency and voltage. A symbol is dark when the maximum induced velocity exceeds the unit diffusion velocity $u_{\mathrm{diff}}$. The vertical dashed line corresponds to the RC frequency.

Figure 17 summarizes the electrohydrodynamical responses of electrolytes under oscillatory voltages. Additional 2D simulations are carried out at voltages smaller than ΔV=180, namely ΔV=40,75,110 and 145. All conditions remain the same except for time-step size for computational efficiency when electroconvective instability is weak or not present. For ΔV=40, the time-step size is increased to $\Delta t/\tau_{\mathrm{diff}}=5\times 10^{-7}$, and for ΔV=75 and 110, it is $2\times 10^{-7}$. The time-step size is $10^{-7}$ for ΔV=145. The additional simulations are time advanced and analyzed for the same number of oscillation periods as those with ΔV=180. In Figure 17, dark symbols correspond to those with the maximum instantaneous velocity being greater than the unit diffusion velocity $u_{\mathrm{diff}}$ denoting the onset of electroconvective instability. No efforts are made to precisely define the boundary between two frequencies at which electroconvection takes place, which belongs to future works.

Several observations can be made in Figure 17. As qualitatively shown in Section 4.1.1, electroconvective instability occurs above a critical voltage at a given oscillation frequency. At applied voltages lower than the critical voltage, no sign of electrohydrodynamic coupling is observed, and the maximum velocity is less than $10^{-6}u_{\mathrm{diff}}$. Also, at a given voltage, a critical frequency exists below which electroconvective instability is amplified. As the frequency is further decreased, one expects the system to return to stable 1D states. However, while we capture non-monotonicity of the behavior, this second transition is not studied in this report, mainly due to requirement of prohibitively long simulations in the low frequency limit. Similar frequency–voltage diagrams are given by Olesen et al. [28] and Stout and Khair [24] to delineate the transition from linear to nonlinear responses of electrolytes under external oscillatory voltages. However, Figure 17 shows the onset of electroconvective instability, a consequence of strongly nonlinear responses of electrolytes. Thus, the critical voltages discussed by Olesen et al. [28] and Stout and Khair [24] are well below the critical voltages shown in Figure 17.

### 4.2. Effects of Ion Diffusivity on Electroconvective Instability

As outlined in Section 2, three different values of diffusivity ratio ($D^{-}/D^{+}=1,5$ and 10) are examined for their effects on electroconvection while the maximum voltage is fixed at ΔV=180. The oscillation frequency $\omega_{0}$ is varied in the same range as in Section 4.1. However, the reduced frequency ${\tilde{\omega }}_{0}$ does not necessarily remain the same since $\omega_{\mathrm{RC}}$ is a function of ionic diffusivity.

**Figure 18:**
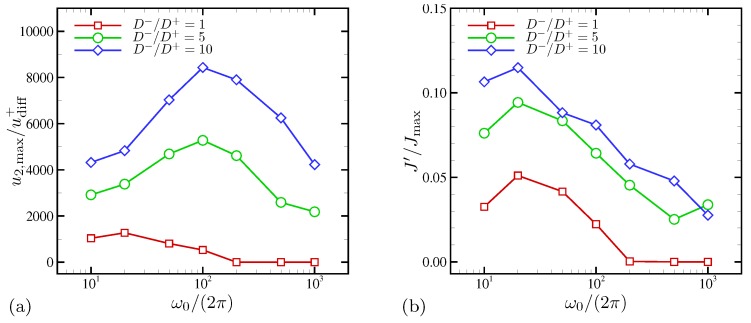
(**a**) Normalized maximum wall-normal velocities and (**b**) normalized surface current density fluctuations in the horizontal direction.

Figure 18 shows the maximum wall-normal velocity (in the unit of the cation diffusion velocity) as a function of $\omega_{0}$. The horizontal velocity (not shown here) shows the same trend. For $D^{-}/D^{+}>1$, the maximum velocities are several times greater than those of $D^{-}/D^{+}=1$ over all frequencies. The peak frequency is $\omega_{0}/\left(2\pi \right)=100$ for both $D^{-}/D^{+}=5$ and 10, higher than $\omega_{0}/\left(2\pi \right)=20$ (or ${\tilde{\omega }}_{0}=0.063$) for $D^{-}/D^{+}=1$.

In Figure 18b, the normalized surface current fluctuations in the horizontal direction are plotted as a function of $\omega_{0}$. For $D^{-}/D^{+}>1$, the fluctuation levels are consistently higher than or comparable to the maximum fluctuation for $D^{-}/D^{+}=1$ over all frequencies examined in this study. This indicates the presence of strong transverse inhomogeneities in surface current fluctuations, linked to electroconvective instability. On the other hand, the equal-diffusivity electrolyte demonstrates the onset of electroconvection at $100≲\omega_{0}/\left(2\pi \right)≲200$, as discussed in Section 4.1.4. The current fluctuations for all cases take the maximum values at the same oscillation frequency, $\omega_{0}/\left(2\pi \right)=20$. Agreement in peak frequencies scaled with cation diffusivity is attributed to cations playing a role of rate-limiting species near the electrode surface where the current is computed.

**Figure 19:**
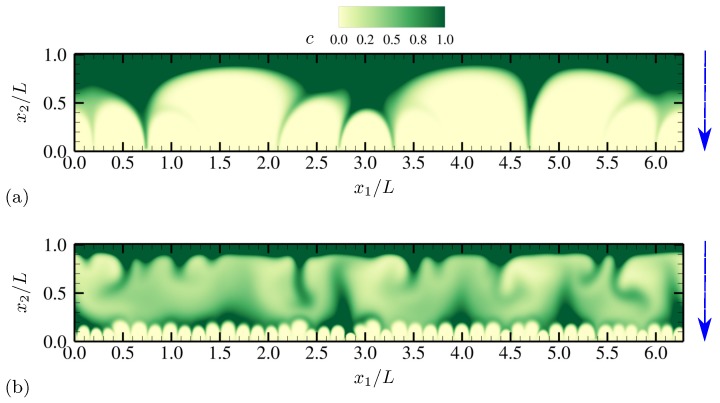
Instantaneous contours of salt concentration *c* at θ=π for (**a**) $\omega_{0}/\left(2\pi \right)=20$ and (**b**) 100. The diffusivity ratio is $D^{-}/D^{+}=10$, and the maximum applied voltage is ΔV=180. The vertical arrows denote the direction of the applied electric field.

**Figure 20:**
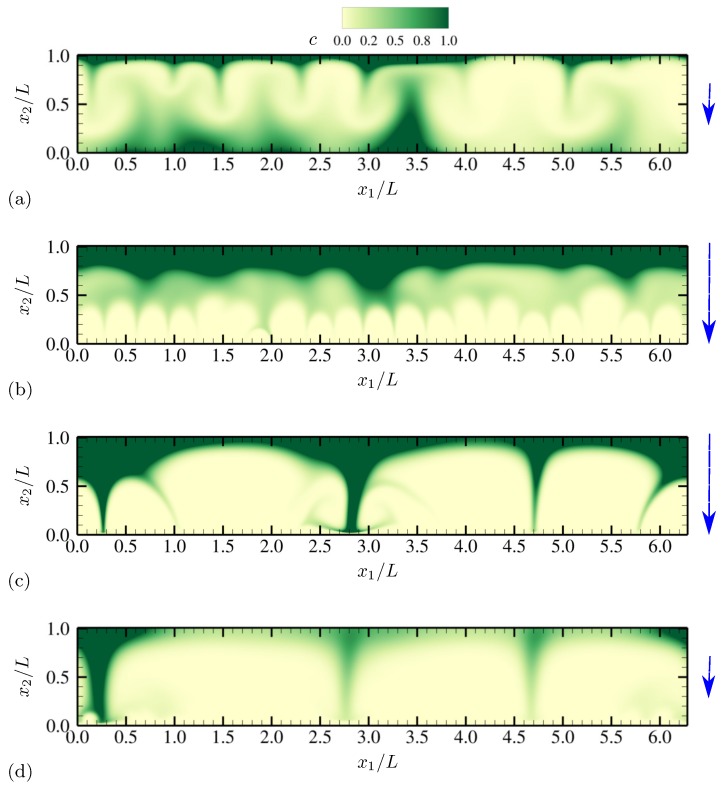
Time series of salt concentration *c* for half an oscillation period at (**a**) θ=0.64π, (**b**) 0.88π, (**c**) 1.12π and (**d**) 1.4π. The vertical arrows denote the direction and relative magnitude (not to scale) of the applied electric field. The diffusivity ratio is $D^{-}/D^{+}=10$.

Figure 19 shows the instantaneous contours of salt concentration for two representative frequencies, $\omega_{0}/\left(2\pi \right)=20$ and 100. The diffusivity ratio is $D^{-}/D^{+}=10$. For both frequencies, distinct asymmetry develops with respect to the channel centerline at $x_{2}/L=0.5$. In Figure 19a, ion-enriched and ion-depleted zones are clearly separated and well distinguished compared with Figure 19b, presumably due to a sufficiently long relaxation time for diffusion. Characteristic large-scale features of size comparable to the channel height are generated via successive agglomeration of smaller structures (see also Figure 20).

**Figure 21:**
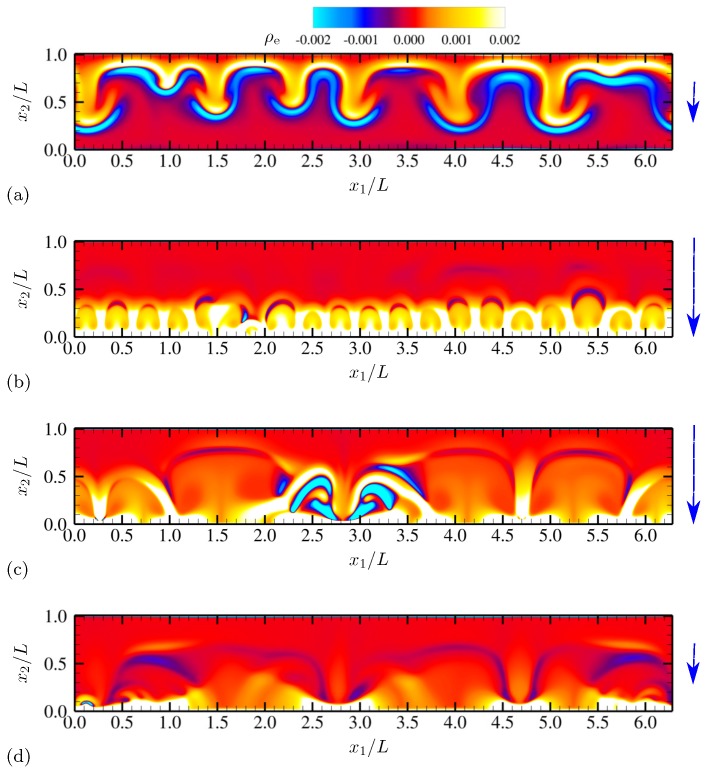
Time series of charge density $\rho_{e}$ for half an oscillation period at (**a**) θ=0.64π, (**b**) 0.88π, (**c**) 1.12π and (**d**) 1.4π. The vertical arrows denote the direction and relative magnitude (not to scale) of the applied electric field. The diffusivity ratio is $D^{-}/D^{+}=10$.

**Figure 22:**
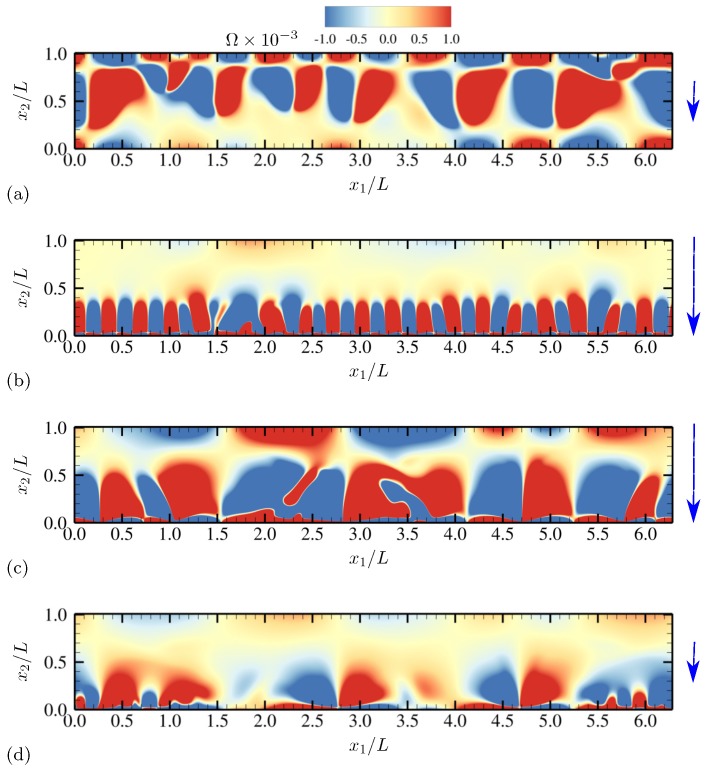
Time series of vorticity Ω for half an oscillation period at (**a**) θ=0.64π, (**b**) 0.88π, (**c**) 1.12π and (**d**) 1.4π. The vertical arrows denote the direction and relative magnitude (not to scale) of the applied electric field. The diffusivity ratio is $D^{-}/D^{+}=10$.

For $\omega_{0}/\left(2\pi \right)=20$, salt concentration, charge density and vorticity during a half oscillation period (between phase angles π/2 and 3π/2) are shown in Figure 20, Figure 21 and Figure 22, respectively. The diffusivity ratio is $D^{-}/D^{+}=10$. As in Figure 3, the bottom electrode remains negatively charged having the minimum voltage $-V_{\max}$ between Figure 20b and Figure 20c. As the voltage difference across the channel increases (from Figure 20a to Figure 20b), anions (having diffusivity 10 times higher than cations) rapidly diffuse and migrate toward the upper electrode, depleting ions at the bottom half of the channel. Due to the much higher anion diffusivity, such process is completed faster than in the equal-diffusivity electrolyte (compare Figure 4b and Figure 21b, for example). Ion-depleted zones further diffuse and form cellular structures (Figure 20c) and almost fill the entire channel (Figure 20d). Figure 22b,c confirm that those structures correspond to the array of counter-rotating vortices, a distinct sign of electroconvective instability. As can be seen in Figure 20c, gaps between adjacent cellular structures contain increasingly high concentration of salt, primarily consisting of counterions (see Figure 21c). Through those gaps, counterions are transported by vortical motions (see Figure 21c and Figure 22c) toward the electrode (at $x_{2}/L=0$ during this time period). The final diffusion processes shown in Figure 20d are preceded by intense breakdown of the gap regions (see Figure 20c and Figure 21c, for example, near $x_{1}/L\approx 2.8$).

**Figure 23:**
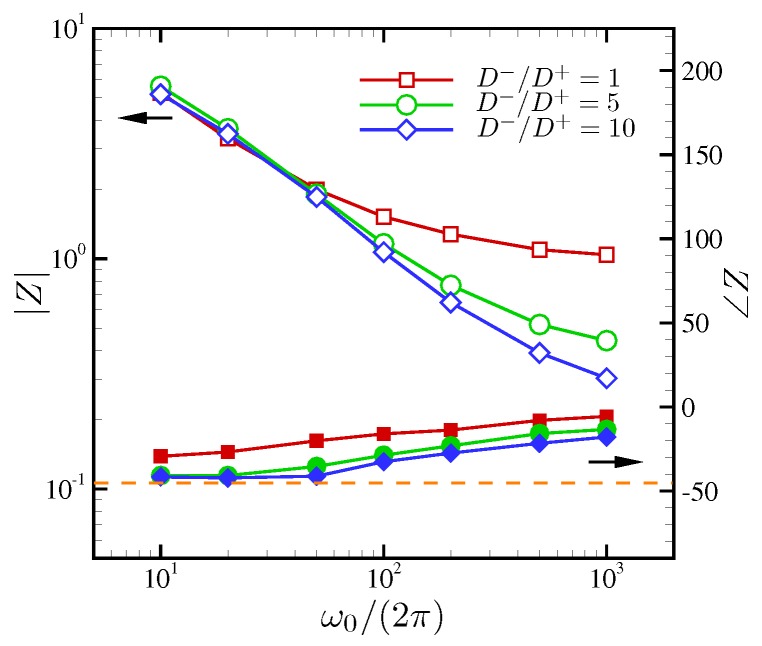
The Bode plot of the overall cell impedance *Z*. The horizontal dashed line corresponds to $∠Z=-45^{\circ }$.

The overall cell impedance is plotted in Figure 23 for all $D^{-}/D^{+}$. As $\omega_{0}$ is reduced, all three curves for |Z| collapse. At such low oscillation frequencies, both cations and anions are given sufficiently long relaxation times. Thus, |Z| is insensitive to diffusivity ratio at $\omega_{0}/\left(2\pi \right)≲50$. The phase angle of the impedance ∠Z asymptotically approaches $-45^{\circ }$, where the characteristic frequency across which the resistor-like behavior of *Z* switches to the capacitor-like one is defined. For all $D^{-}/D^{+}$, *Z* never behaves like a perfect capacitor, an observation that can be explained using the amplified electric current by electroconvection. As the oscillation frequency increases, the equal-diffusivity electrolyte ohmically transfers electric current (|Z|→1 and $∠Z\to 0^{\circ }$). However, at the same frequency limit, significant discrepancies in |Z| are observed for $D^{-}/D^{+}>1$. The impedance magnitude decreases up to 3.4 times for $D^{-}/D^{+}=10$, implying the corresponding decrease in resistivity of electrolyte and increase in electric current.

**Figure 24:**
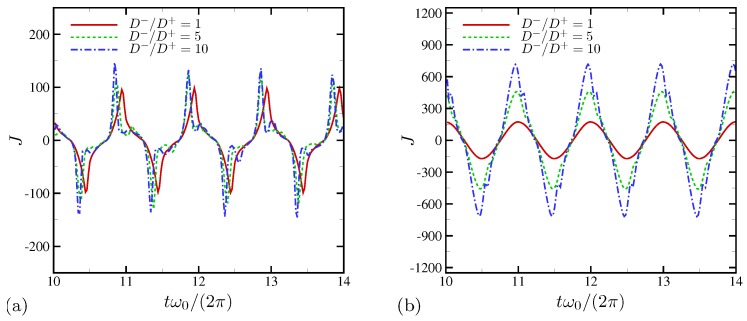
Surface current per unit area for (**a**) $\omega_{0}/\left(2\pi \right)=20$ and (**b**) 1000.

The surface current density for all $D^{-}/D^{+}$ is computed and shown for 4 oscillation periods in Figure 24. For $\omega_{0}/\left(2\pi \right)=20$ (Figure 24a), the increased diffusivity ratio causes a phase-lead of the current peaks (see ∠Z in Figure 23), presumably a direct impact of anion diffusing much faster than cation. The maximum surface current also increases by up to 50%. However, the total current density remains nearly the same, consistent to the same impedance amplitude for all $D^{-}/D^{+}$ at $\omega_{0}/\left(2\pi \right)=20$, as shown in Figure 23. In Figure 24b, the results for $\omega_{0}/\left(2\pi \right)=1000$ are shown for 4 oscillation periods as well. The amount of phase-lead is substantially reduced, consistent to Figure 23. There is significant amplification in the maximum surface current by 2.7 times ($D^{-}/D^{+}=5$) and 4.2 times ($D^{-}/D^{+}=10$). This is also consistent to the observation made at $\omega_{0}/\left(2\pi \right)=1000$ in Figure 23.

**Figure 25:**
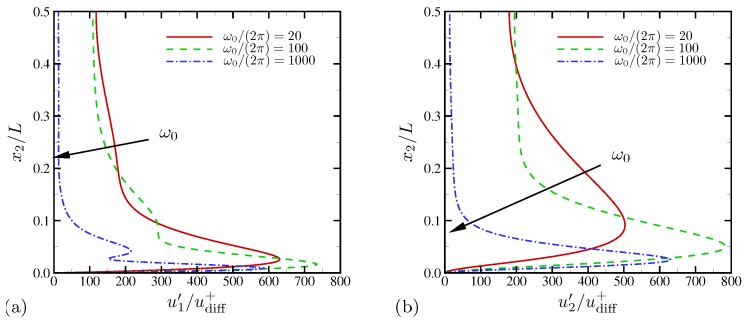
Rms velocity fluctuations versus wall normal coordinate for (**a**) horizontal and (**b**) wall-normal velocities. The diffusivity ratio is $D^{-}/D^{+}=10$.

In Figure 25a, the RMS profiles of horizontal velocity fluctuations with respect to time and the horizontal direction are shown as a function of wall-normal distance. Over all frequencies examined in this study, predominant RMS peaks are observed very near the electrode surface. A broader, secondary peak is found away from the primary peak for all three frequencies. At the highest frequency $\omega_{0}/\left(2\pi \right)=1000$, the centerline fluctuation is $20u_{\mathrm{diff}}^{+}$, higher than the centerline value for $\omega_{0}/\left(2\pi \right)=100$ (or ${\tilde{\omega }}_{0}=0.314$) in Figure 7 where electroconvection is reported for equal-diffusivity electrolytes (see Figure 16 or Figure 17). Overall, the RMS profiles do not show consistent changes as frequency is varied. In the bulk fluid, frequency increase is correlated with reduced RMS fluctuation. However, the primary peaks do not follow the same trend, and the intermediate frequency $\omega_{0}$ leads to the highest fluctuation peak. For wall-normal velocities shown in Figure 25b, the intermediate frequency $\omega_{0}/\left(2\pi \right)=100$ also has the largest peak near the electrode surface. Except for $\omega_{0}/\left(2\pi \right)=1000$, the centerline level of wall-normal velocity fluctuations is consistently high. Figure 25 shows that, for $D^{-}/D^{+}=10$, electroconvection is triggered for all frequencies but rather confined to near-wall regions at the highest frequency, $\omega_{0}/\left(2\pi \right)=1000$.

**Figure 26:**
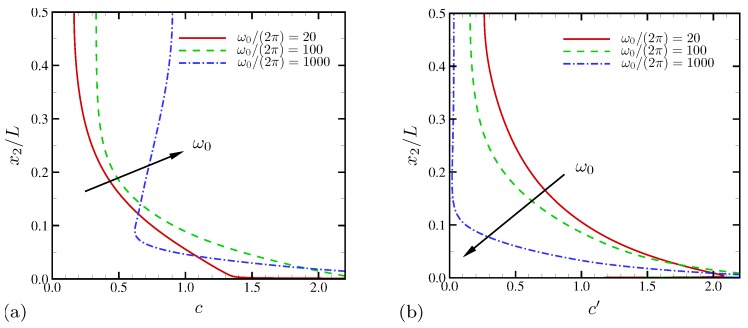
The wall-normal profiles of (**a**) time-averaged salt concentration and (**b**) salt fluctuation RMS. The diffusivity ratio is $D^{-}/D^{+}=10$.

Time-averaged salt concentration is shown in Figure 26a for $D^{-}/D^{+}=10$. Similar to velocity statistics in Figure 25, frequency increase does not result in consistent changes of salt at every wall-normal location. At $\omega_{0}/\left(2\pi \right)=20$ and 100, mean salt distribution shows monotonic decrease toward the bulk fluid. Compared with Figure 8 for the equal-diffusivity electrolyte, excessive bulk salt depletion (below 40%) is observed. However, the bulk salt is mildly depleted for $\omega_{0}/\left(2\pi \right)=1000$. Instead, near the electrode (at $x_{2}/L\approx 0.08$), strong salt depletion is observed. The profiles of RMS salt fluctuations (with respect to time and the horizontal direction) are shown in Figure 26b. As $\omega_{0}$ increases, salt fluctuations rapidly drops. For $\omega_{0}/\left(2\pi \right)=1000$, the fluctuation levels in the bulk fluid becomes negligibly small for $x_{2}/L≳0.1$, confirming that electroconvective instability at this oscillation frequency is mostly confined to near-electrode regions.

**Figure 27:**
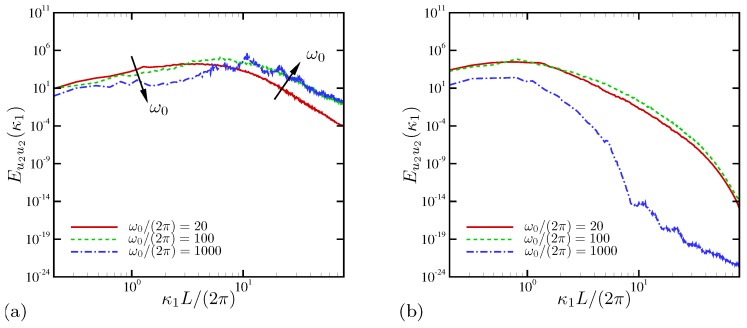
Spatial power spectral density of wall-normal velocity at (**a**) $x_{2}/L=0.03$ and (**b**) 0.5. The diffusivity ratio is $D^{-}/D^{+}=10$.

For $D^{-}/D^{+}=10$, the spatial PSD of wall-normal velocity are shown in Figure 27 at $x_{2}/L=0.03$ (approximately the ESC region) and 0.5 (the channel centerline), respectively. Near the electrode surface, broadbanded spectra over all wavenumbers are observed for all oscillation frequencies, as can be seen in Figure 27a. Large- and small-scale motions respond to frequency increase in the opposite way (note that for $D^{-}/D^{+}=1$ all scales respond in a consistent manner as frequency changes; see Figure 10a). Close to the electrode, lower external frequencies are more effective in enhancing large-scale fluctuations, while high frequencies generally amplify small-scale motions. At $\omega_{0}/\left(2\pi \right)=1000$, mild discrete peaks are found at $\kappa_{1}L/\left(2\pi \right)=10$, 20, and 30, respectively, consistent to the relatively weaker electroconvective instability observed at that frequency. As shown in Figure 27b, the responses of electrolytes at the centerline are quite different and do not show monotonic behavior with respect to the oscillation frequency. At lower frequencies, power spectra are still broadbanded at the centerline, whereas fluctuation energy rapidly decays for $\omega_{0}/\left(2\pi \right)=1000$, consistent to Figure 25b. The PSD of horizontal velocity show nearly the same trend, and thus they are not shown here.

**Figure 28:**
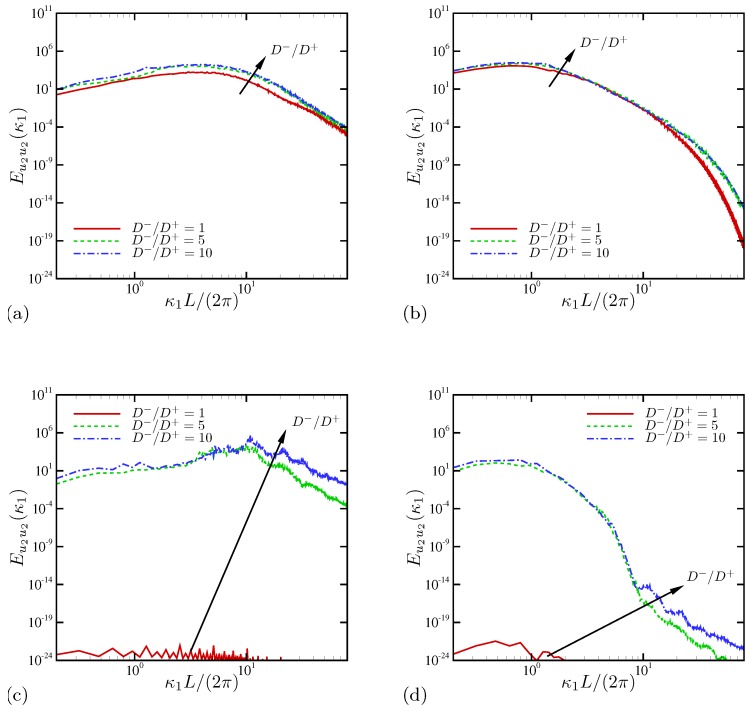
Spatial power spectral density of wall-normal velocity: (**a**) $\omega_{0}/\left(2\pi \right)=20$ and $x_{2}/L=0.03$; (**b**) $\omega_{0}/\left(2\pi \right)=20$ and $x_{2}/L=0.5$; (**c**) $\omega_{0}/\left(2\pi \right)=1000$ and $x_{2}/L=0.03$; (**d**) $\omega_{0}/\left(2\pi \right)=1000$ and $x_{2}/L=0.5$

Figure 28 summarizes the effects of diffusivity ratio on spatial PSD of wall-normal velocity. In general, increasing the diffusivity ratio always promotes velocity fluctuation levels over all length scales. At a low AC frequency (Figure 28a,b), $D^{-}/D^{+}$ has marginal impacts on $E_{u_{2}u_{2}}$ near the electrode and at the centerline. As the oscillation frequency is increased (Figure 28c,d), distinct effects of $D^{-}/D^{+}$ appear primarily at small scales, $\kappa_{1}L/\left(2\pi \right)≳10$, irrespective of wall-normal positions. At intermediate frequencies (not shown), near-electrode responses to varying $D^{-}/D^{+}$ are similar to those of low frequencies (Figure 28a), presumably due to smaller length scales there, whereas the centerline responses are pronounced similar to Figure 28d at $\kappa_{1}L/\left(2\pi \right)≳1$.

Analyses so far suggest that the role of unequal diffusivity includes significant promotion of electroconvective instability, increase in the maximum current over all frequency range examined in this study and amplified total current at the ohmic regime. The effects of unequal diffusivity are more pronounced at high oscillation frequencies and at smaller scales (≲0.1L). At low AC frequencies, asymmetry in ionic diffusivity increases spatial PSD over all scales; however, further increasing the diffusivity ratio does not have much impact.

## 5. Conclusions and Discussions

Electroconvective instability under alternating current (AC) electric fields is discovered for the first time and characterized using solutions of 2D direct numerical simulation (DNS) of aqueous binary electrolytes bounded by parallel inert blocking electrodes. In addition to voltage magnitude and oscillation frequency, the effects of ionic diffusivity on electroconvection are examined. Similar to electroconvection under direct current (DC) electric fields, strong coupling between ion transport and hydrodynamics is observed under the conditions where AC electroconvection is allowed to occur. This is manifested by the formation of the arrays of counter-rotating vortical structures periodically created and destroyed near the flat inert electrode surfaces. Broadbanded power spectral density (PSD) is observed in spatiotemporal spectral analysis of average salt and velocity fields, demonstrating strong nonlinearity necessary to trigger and sustain electroconvective instability. It is found that a critical maximum voltage is a function of oscillation frequency. Below such critical voltages, the classical asymptotic structures of electric double layer (EDL) and extended space-charge (ESC) layer are recovered and show good agreement between the 1D theory and the current DNS prediction. A critical oscillation frequency at which AC electroconvection occur is also examined. Compared with the resistor–capacitor (RC) frequency of the equivalent AC circuit corresponding to the flat-electrode setup, the critical frequency is much lower than the characteristic RC frequency. The optimal responses (defined using the maximum induced instantaneous velocity or the surface current density fluctuation) occur well below the RC frequency (a few percent of the RC frequency). Salt depletion is much more severe than what 1D theory predicts. Increase in the maximum current is accompanied with strong electroconvection, while at frequencies higher than the critical frequency, the Debye layer charges incompletely and the 2D eletrolyte behaves essentially like a 1D system with little or no perturbation in the bulk solution. Interestingly, for the considered case of $\lambda_{D}=10^{-3}L$, the inner-layer structures at $x_{2}/L≲0.03$ (where *L* is the distance between the two electrodes) are insensitive to electroconvection and remain 1D. The system-level responses estimated using the overall cell impedance reveals important changes in electrolyte once electroconvection takes place. The eletrolyte below the critical frequency never behaves like a perfect capacitor and instead allows increased electric current across the microfluidic channel. The effects of ionic diffusivity on electroconvective instability are more complex and not monotonic as parameters such as the maximum voltage difference and the oscillation frequency are varied. In general, asymmetry in diffusivity facilitates electroconvective instability. At all external AC frequencies examined in this study, electroconvection is reported. Electric current is amplified, in particular, at high frequency regimes, which is opposite to the results from a symmetric electrolyte. Also, high anion diffusivity has stronger impacts on smaller-scale motions (≲0.1L).

Oftentimes, realistic microfluidic devices are 3D. The presence of the third spatial direction may cause additional complexities and challenges to the analysis of electroconvective motions. However, Druzgalski and Mani [12] reports that statistics of 2D and 3D electrokinetic systems under the external DC voltages do not show significant qualitative differences under highly chaotic regimes, as can be seen in their Figure 8 and Figure 9, by which some of the important conclusions made by the present study can be, at least qualitatively, extended to 3D systems subject to oscillatory voltages.

Similar to turbulence simulation, DNS applied to electrokinetic flows offers very detailed space- and time-resolved dynamics of ionic and hydrodynamic transports, useful to understand fundamental physics and validate theoretical models. However, its direct extension to realistically high voltage and diffusivity ratio is not straightforward due to several challenges in modeling and numerical method. In particular, the presence of very thin EDLs near ion-selective surfaces increases computational costs significantly when DNS is employed to resolve the EDL structure. In this study, 13 cells are used to represent the nominal EDL thickness $\lambda_{D}/L=10^{-3}$. As pointed out in Section 2, this value is sufficiently small to represent some microfluidic configurations. However, realistic nondimensional Debye layer thickness can often become one to two orders-of-magnitude smaller than $10^{-3}$, which requires to reduce the minimum grid spacing severely and thus results in a tremendously large number of grid points (also prohibitively small computational time-step size) in 3D setups. One of the findings in this study suggests that by adequately modeling the inner layer dynamics (which is insensitive to electroconvection in the outer layers), such drastic increases in computational costs can be accommodated. Ideas similar to wall-layer modeling of turbulent boundary layers [34] can be adapted for electrokinetic flow simulations.

In practice, the maximum diffusivity ratio of multi-species electrolytes can become as high as 1000 (due to electrons in plasmas, for example). Thus, a single ionic species having a very large diffusivity may severely limit the computational time-step size, determined by its diffusion time scale. The second-order backward Euler time advancement used in this study is stable for configurations having modest diffusivity ratios (for example, $D^{-}/D^{+}=10$). However, for realistically high diffusivity ratios, more robust or even fully-implicit temporal discretization appears to be necessary to have a sufficiently large computational time-step size. This can be critical to simulating AC electroconvection where its optimal frequency at which electroconvective instability is most pronounced is well below the characteristic frequency such as the RC frequency.

As discussed in Section 2, the current formulation does not include the compact Stern layer near electrodes nor adopts the modified Poisson–Nernst–Planck (PNP) models accounting for the steric effects. In the 1D setup, Olesen et al. [28] provides extensive studies to characterize the effects of the two models on weakly and strongly nonlinear responses of electrolyte under oscillating external voltages. One of their key findings is that significant potential drops occur across the Stern layer, and the steric inclusion limits the unrealistic crowding of ionic species near electrodes. Thus, concentration gradient is substantially reduced, and nonlinear responses are attenuated compared with the formulation based on the classical Poisson–Boltzmann distribution at the same applied voltage. Although the present study does not take into account such effects, it is expected that including such models delay the onset of electroconvection or at least weakens its magnitude, which can be verified by future studies.

## Figures and Tables

**Table 1:** Dimensionless parameters and their descriptions.

Dimensionless Parameters	Descriptions
$\kappa =\varepsilon V_{T}^{2}/\left(\mu D^{+}\right)$	electrohydrodynamic coupling constant
$\epsilon =\lambda_{D}/L$	nondimensional Debye screening length
$\mathrm{Sc}=\mu /(\rho D^{+})$	Schmidt number
$\Delta V=V_{\max}/V_{T}$	nondimensional maximum voltage
$\omega_{0}$	nondimensional oscillation frequency
ratio of ionic diffusivities	$D^{-}/D^{+}$

## Supplementary Materials

The following are available online at https://www.mdpi.com/2072-666X/10/3/161/s1, Video S1: Time series of salt concentration for a single oscillation period; Video S2: Time series of charge density for a single oscillation period; Video S3: Time series of vorticity for a single oscillation period.
