# Supplementary figures and images for: Characterization of Chaotic Electroconvection near Flat Inert Electrodes under Oscillatory Voltages
Source: Micromachines (Basel). 2019 Feb 26;10(3):161. doi: 10.3390/mi10030161 (PMC6470596; doi:10.3390/mi10030161)

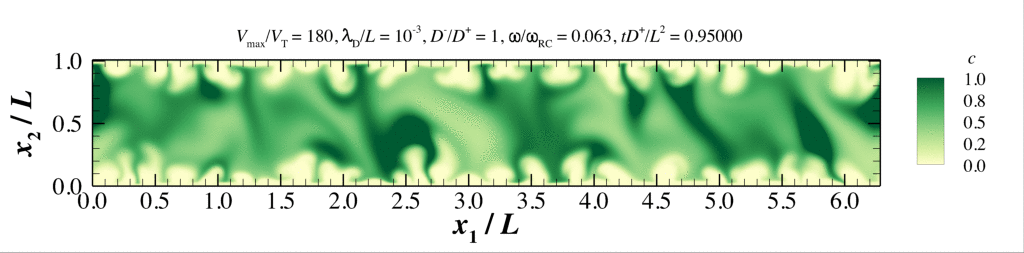

Supplement: Supplementary file 1 [file micromachines-10-00161-s001.zip › micromachines-448735 suppl - final/Video S1.gif]

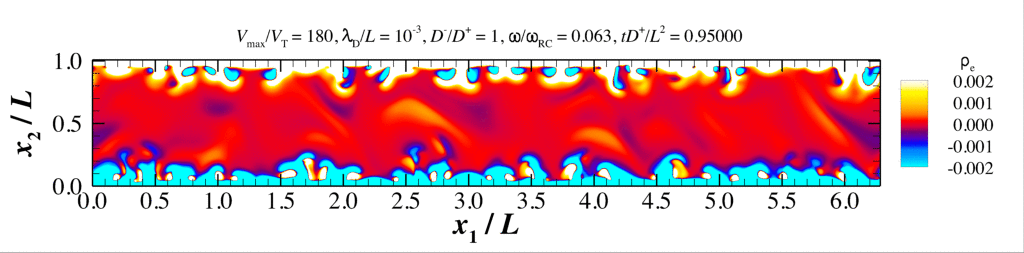

Supplement: Supplementary file 1 [file micromachines-10-00161-s001.zip › micromachines-448735 suppl - final/Video S2.gif]

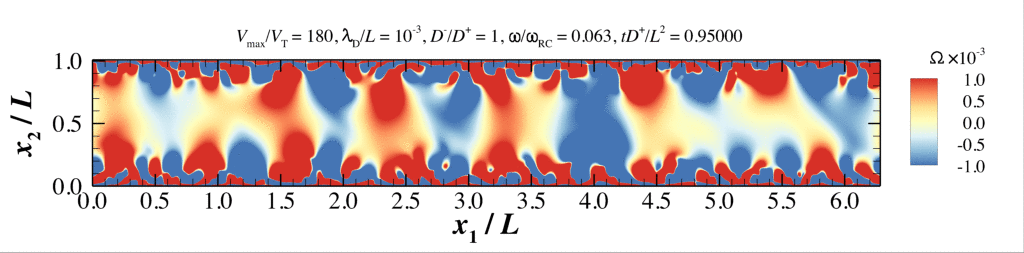

Supplement: Supplementary file 1 [file micromachines-10-00161-s001.zip › micromachines-448735 suppl - final/Video S3.gif]
